# Effects of Four Highland Barley Proteins on the Pasting Properties and Short-Term Retrogradation of Highland Barley Starch

**DOI:** 10.3390/molecules29061211

**Published:** 2024-03-08

**Authors:** Ran Lin, Mengzi Nie, Jiaxin Li, Aixia Wang, Xue Gong, Fengzhong Wang, Lili Wang, Liya Liu, Bin Dang, Xijuan Yang, Xijun Lian, Li-Tao Tong

**Affiliations:** 1 Tianjin Key Laboratory of Food Biotechnology, School of Biotechnology and Food Science, Tianjin University of Commerce, Tianjin 300134, China; linran1669120430@163.com; 2 Institute of Food Science and Technology, Chinese Academy of Agricultural Sciences/Key Laboratory of Agro-Products Processing, Ministry of Agriculture, Beijing 100193, China; niemengzi315@163.com (M.N.); lijiaxin0717@foxmail.com (J.L.); 8210119600@caas.cn (A.W.); xuegong1214@163.com (X.G.); wangfengzhong@caas.cn (F.W.); wlland2013@163.com (L.W.); liuliya1218@163.com (L.L.); 3 Qinghai Tibetan Plateau Key Laboratory of Agric-Product Processing, Qinghai Academy of Agricultural and Forestry Sciences, Xining 810016, China; 2008990019@qhu.edu.cn (B.D.); 2007990025@qhu.edu.cn (X.Y.)

**Keywords:** highland barley starch, pasting properties, short-term retrogradation, protein–starch interaction

## Abstract

This study evaluated the effects of four highland barley proteins (HBPs), namely, albumin, globulin, gliadin and glutenin, on the short-term retrogradation of highland barley starch (HBS). The findings reveal that HBPs could reduce the viscosity, storage modulus and hardness of HBS, with albumin and globulin showing more prominent effects. Furthermore, with the addition of HBPs, the loss tangent (tan δ) of HBS loss increased from 0.07 to 0.10, and the enthalpy of gelatinization decreased from 8.33 to 7.23. The degree of retrogradation (DR%) of HBS was 5.57%, and the DR% decreased by 26.65%, 38.78%, 11.67% and 20.29% with the addition of albumin, globulin, gliadin and glutenin, respectively. Moreover, the relative crystallinity (RC) and the double helix structures were inhibited with the HBPs’ incorporation. Meanwhile, the HBPs also could inhibit water migration and improve the structure of HBS gels. In summary, HBPs could inhibit the retrogradation behavior of HBS, which provides new theoretical insights for the production studies of highland barley foods.

## 1. Introduction

Highland barley (HB), which grows mainly in northwestern and southwestern China, is the traditional staple food of the Tibetan people [1]. In recent years, HB has gained attention for its ability to reduce the incidence of illnesses such as hyperlipidemia, hypertension and diabetes mellitus [2]. The primary ingredient of HB grains is starch (47.9–79.0%), which is closely linked to the processing of HB products [3]. However, this retrogradation behavior of starch usually has some adverse effects, such as causing starch-rich foods to spoil, leading to significant wastage and thus causing serious challenges for food manufacturers [4]. Therefore, some useful methods need to be adopted to improve the performance of starch and slow down retrogradation.

Starch and protein are two important components in food, and their interaction affects the texture, stability and digestibility of food [5]. Previous studies have shown that protein can significantly affect the gelatinization, rheological properties and texture of starch while avoiding harmful components brought about by chemical modification [6]. Some researchers have observed that protein could attach to the starch granule surface during gelatinization, limiting water diffusion and resulting in lower pasting viscosity and a higher pasting temperature, thereby reducing and delaying starch particle expansion [7]. According to Zhang et al. [8], the incorporation of rice protein weakened the conversion of bound to free water, reduced starch molecular crossing links and limited ordered structure formation. In addition, it has been shown that exogenous proteins can form non-covalent bonds (such as hydrophobic interactions and hydrogen bonds) and covalent bonds with starch during heating or form physical barriers, thus affecting the retrogradation of starch [9]. Therefore, exploring more protein–starch interactions has far-reaching implications for improving food quality.

Highland barley protein (HBP) is an important nutrient component second to starch in HB, and its content accounts for 8.20–20.80% of HB [1]. HBP can be classified into four isolated proteins based on solubility: albumin, globulin, gliadin and glutenin, which account for 12.95%, 12.73%, 16.96% and 47.83% of the total protein content, respectively [10]. Studies have shown that the addition of recombinant gluten composed of different ratios of glutenin/gliadin can effectively inhibit wheat starch retrogradation [11]. This demonstrates the potential ability of different types of isolated proteins to improve starch retrogradation. The effect of the four highland barley proteins (HBPs) on HBS retrogradation, of which proteins have more significant inhibitory effects, and the protein–starch interaction mechanisms are currently unknown, which stimulates the research in this field.

Starch granules swell and rupture when heated in water, and precipitated amylose and amylopectin can be recombined into an ordered system when cooled, which is called retrogradation [12]. Retrogradation, divided into short- and long-term retrogradation, is an uninterrupted process caused by amylose and amylopectin, respectively [13]. Currently, the majority of research on starch retrogradation has been focused on long-term retrogradation, yet in reality, short-term retrogradation is more closely related to our daily diet. Short-term retrogradation, which typically occurs within a few hours, primarily involves the aggregation of amylose to form a crystal nucleus [14,15]. Studies have shown that short-term retrogradation plays a crucial role in influencing the early hardness, viscosity and digestibility of starch-based foods [16]. Meanwhile, despite being a highly valuable crop, there has been relatively limited exploration related to HB. Therefore, it is valuable to investigate the impact of HBPs on the short-term retrogradation of HBS. This can not only realize the value-added application of highland barley, but also provide a theoretical basis for guiding daily diet. We speculated that HBPs could act as a retrogradation inhibitor to hinder amylose rearrangement and inhibit amylose recrystallization, thereby inhibiting short-term retrogradation of HBS.

In this study, four types of HBPs (albumin, globulin, gliadin and glutenin) were extracted from HBP and used as additives to prepare HBS gels. The effects of HBPs on the gelatinization properties of HBS were systematically explored through RVA testing, rheological testing and DSC testing. Additionally, the influence of the four proteins on the short-term retrogradation of HBS was evaluated by studying the water migration, hardness, short- and long-range order and microstructure of the starch gels. Furthermore, the mechanism of the protein–starch interaction was explored. The results of the study will further inform and guide the production and quality improvement of HB.

## 2. Results and Discussion

### 2.1. Pasting Properties

**Figure 1:**
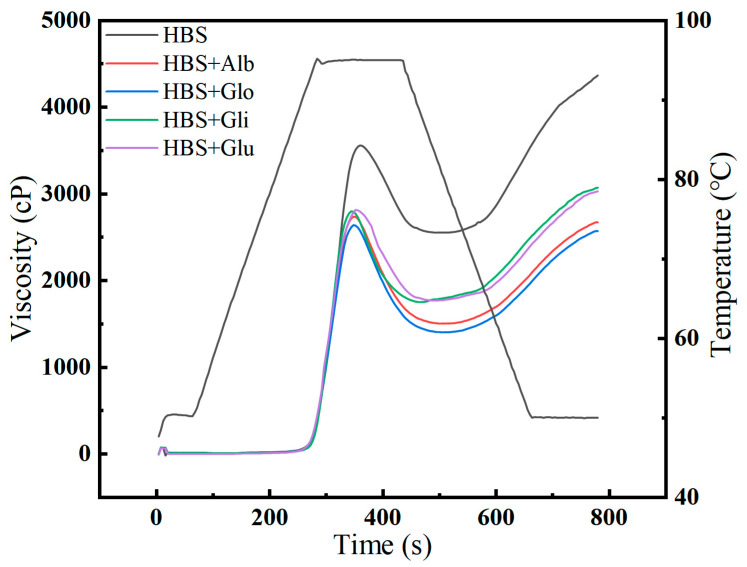
Pasting curves of HBS and HBS/HBPs mixtures. Alb: albumin; Glo: globulin; Gli: gliadin; Glu: glutenin.

During the process of starch gelatinization, starch granules absorb water and swell, resulting in a transition of their molecular structure from ordered to disordered, along with the destruction of the crystal structure. This alteration affects the physical properties and viscosity of the starch [17]. As shown in Figure 1, the pasting curves of HBS and HBS/HBPs mixtures are presented, and the pasting properties are summarized in Table 1. The addition of HBPs to HBS resulted in a decrease in the peak viscosity (PV), trough viscosity (TV), final viscosity (FV), breakdown (BD) and setback (SB) values, while the pasting temperature (PT) increased. Notably, the peak viscosity (PV) serves as an indicator of the degree of expansion of starch granules during the gelatinization process. Compared to the blank group, the PV values decreased by 26.24%, 29.23%, 22.49% and 19.86% with the addition of the albumin, globulin, gliadin and glutenin, respectively, implying that HBPs significantly (*p* < 0.05) inhibited the swelling of starch granules, which is similar to previous reports that pea protein reduced the PV of cassava and corn starches [18]. The unique properties of these four proteins—the water solubility of albumin enabling it to compete with starch for more water molecules, the ability of globulin to reduce the total water absorption of starch granules during gelatinization, gliadin’s formation of a physical barrier on the surface of starch granules when heated, and the creation of hydrophobic polymers by glutenin through covalent bonds—respectively lead to the inhibition of starch gelatinization and subsequent decrease in peak viscosity (PV) values [11,19,20]. In addition, the effect of albumin and globulin was more significant than gliadin and glutenin when the same amount of protein was added. This result may be related to the difference in the relative molecular weights of the proteins: the molecular weight of albumin and globulin is generally 12–60 kDa, gliadin is generally 30–63 kDa and glutenin is generally 40–300 kDa. Renzetti et al. found that smaller protein molecules reduced the viscosity of the pasted starch more than larger protein molecules [21].

The spread between PV and TV is the BD viscosity, which indicates the damage extent of starch granules during the pasting process. Generally, the lower the value, the better the shear resistance of the granules [22]. The results show that all four proteins reduced the BD viscosity at the same addition amount, indicating that they all could inhibit the expansion and rupture of starch [23]. Ribotta et al. [18] indicated that the BD viscosity of cassava starch was reduced after adding enzyme-treated pea protein. The “denatured protein’s gel matrix” formed during gelatinization was reported to provide mechanical sustenance for the starch granules, inhibit their maximal expansion and limit their rupture, leading to a lower amount of leached amylose [24].

The SB viscosity is closely associated with the level of amylose recrystallization and rearrangement during cooling and could be used to represent the extent of short-term retrogradation [25]. Similar to the BD results, the addition of HBPs decreased the SB viscosity of HBS from 1870 cp to 1168, 1048, 1430 and 1366 cp, respectively, which showed that the HBPs had a particular capacity to inhibit the short-term retrogradation of HBS. This may be due to proteins interacting with leached amylose through hydrogen bonds, hydrophobic forces and electrostatic adherence, resulting in reduced interaction with the amylose [26,27]. In summary, HBPs could limit the swelling and rupture of starch granules, reduce the amount of leaching of amylose and inhibit the short-term retrogradation of starch by interacting with amylose during heat treatment.

PT refers to the temperature at which the viscosity starts to increase during the gelatinization process, and its increase is directly related to the decrease in free water content within the system [28]. The addition of protein can affect the distribution of water, ultimately altering the interactions between water molecules and other components, thereby leading to an increase in PT [9].

### 2.2. Dynamic Viscoelastic Properties

**Figure 2:**
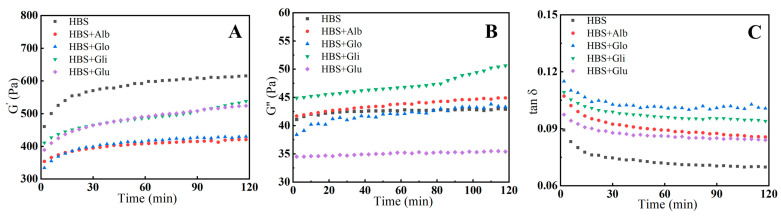
Changes in the storage moduli (G′) (**A**), G″ (**B**) and tan δ (**C**) of HBS and HBS/HBPs gels during an isothermal time sweep step at 4 °C for 2 h. Alb: albumin; Glo: globulin; Gli: gliadin; Glu: glutenin.

The increase in G′ is primarily related to the formation of a three-dimensional gel network through the aggregation of amylose during the early stages of retrogradation [29]. Therefore, G′ can be used as a metric to assess the short-term retrogradation of amylose. The G″ characterizes the viscous behavior of the temporary molecular network structure formed by the gelatinized starch. As observed in Figure 2A,B, the growth of the G′ value initially occurred at a relatively fast rate, followed by a slower rate, while the growth of the G″ value remained very slow throughout, with no plateau region appearing in either of them during the experiment. This suggests that the leached amylose, under storage conditions of 4 °C, first aggregated rapidly and then proceeded to a slow rearrangement, resulting in a gradual hardening of the gel samples [30]. The presence of HBPs reduced the G′ values compared to HBS, and the increment in G′ comparatively reduced with the extension of storage time. This decreasing tendency demonstrates that HBPs inhibited the gelation of amylose, leading to a decrease in elastic properties, which also implies that the recrystallization of the starch was suppressed. It could be inferred that the decrease in G′ values occurred since the interaction between HBPs and amylose reduced the aggregation of amylose [31]. Furthermore, the G′ values of the albumin and globulin groups were the lowest among all the samples, indicating that they inhibited retrogradation more effectively than gliadin and glutenin. It has been shown that albumin and globulin are more likely to compete with starch for water and interact with amylose than gliadin and glutenin, which can reduce the precipitation of amylose and weaken the three-dimensional network structure of the gel [19,20].

The change in tan δ (G″/G′), as shown in Figure 2C, is an important indicator of the relative contribution of viscous and elastic components to viscoelastic properties [13]. The results indicate that the tan δ value gradually decreased as time increased, which means that all the samples underwent retrogradation. Meanwhile, the HBS/HBPs mixtures had higher tan δ values than HBS, suggesting that the composite gel contained relatively fewer elastic fractions and more viscous fractions. This means that the HBPs inhibited the rearrangement of amylose, resulting in a looser gel structure. In addition, the tan δ values increased from 0.089 and 0.084 when adding gliadin and glutenin to 0.093 and 0.103 when adding albumin and globulin after 2 h, suggesting that the addition of albumin and globulin made the gel networks more disordered, which is in agreement with the conclusion for G′.

### 2.3. Thermal Properties

**Figure 3:**
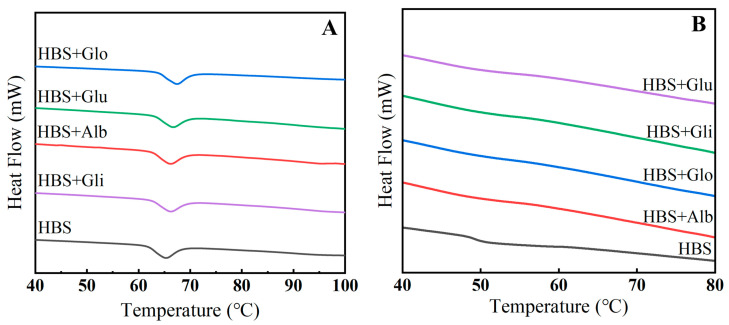
Gelatinization (**A**) and retrogradation (**B**) curves of HBS and HBS/HBPs mixtures. Alb: albumin; Glo: globulin; Gli: gliadin; Glu: glutenin.

The thermal characteristics test, as shown in Figure 3A, further explores the impact of HBPs on the short-term retrogradation of HBS. It was observed that all the samples exhibited an endothermic peak within the range of 60~70 °C, and the addition of HBPs caused the endothermic peak to shift towards higher temperatures. This was due to the redistribution of water caused by the addition of protein, which delayed the gelatinization of starch [5].

Meanwhile, as shown in Table 2 and Figure 3B, the onset temperature (T_O_), peak temperature (T_P_) and conclusion temperature (T_C_) of the HBS/HBPs mixtures were higher than those of HBS, which means that the HBPs restrained the expansion of starch granules during the pasting process. Studies have shown that the increase in gelatinization temperature is caused by the interaction between charged amino acids and starch chains, and the effect depends on the absolute value of the net charge [32]. In addition, this interaction altered the overall net charge of the starch suspension, leading water molecules to attach to protein chains due to increased repulsive forces, which reduced the amount of mobile water in the system, thereby impeding the expansion of starch granules [33]. The ΔH_g_ represents the amount of energy required for pasting to occur in starch granules, and it has been shown that enthalpy changes are mainly related to the rate of temperature increase, the degree of hydration, the moisture content and the disruption of the crystalline order [34]. In our study, the ΔH_g_ of the HBS/HBPs mixtures was significantly reduced after the addition of HBPs (*p* < 0.05), which could be attributed to the change in the effective water content for hydration during dissolution at the same heating rate, which accelerated the interaction of the swollen starch granules with the proteins, resulting in the starch of the mixed system being more easily pasted than the pure starch system [35]. In addition, the decrease of ΔH_g_ generally means a decrease in the double helix of amylose, as well as a reduction in the number of crystals [36]. Meanwhile, ΔH_r_ provides an accurate measure of the energy conversion during the endothermic event of crystal melting, and a larger value indicates a higher degree of starch crystallization and starch retrogradation [37]. The results indicate that the addition of HBPs significantly reduced the ΔH_r_ of HBS (*p* < 0.05), implying the formation of fewer ordered crystals in the system. In addition, after the addition of four HBPs, the retrogradation rate of HBS (5.57%) decreased by 26.65%, 38.78%, 11.67% and 20.29%, respectively, which once again demonstrated the inhibition of HBPs on HBS retrogradation, as well as that of the albumin and globulin being more significant. This was primarily due to the competitive inhibition of water between albumin/globulin and starch, which suppressed the amylose leaching and subsequently reduced the formation of ordered crystals during short-term storage. The inhibition of gliadin and glutenin on retrogradation was mainly due to their formation of aggregates that hindered amylose rearrangement.

### 2.4. LF-NMR Analysis

The water distribution and migration in the gel are also important parts of retrogradation, and low-field NMR can quickly and easily reveal the moisture movement in the retrogradation process [29]. The relaxation time distributions (T_2_) and area fractions (A_2_) of the HBS and HBS/HBPs gels are shown in Table 3. We can see that water molecules with three different mobility levels were distinguished. T_2_ can be divided into T_21_ (0.6–3.0 ms), T_22_ (5–80 ms) and T_23_ (>100 ms), which represent water molecules with different mobility, and the corresponding peak area ratios are labelled as A_21_, A_22_ and A_23_, respectively. The lower T_2_ values indicate that the starch molecules are more tightly bound to the water molecules, thereby reducing the mobility of the water [37]. The peaks T_21_, T_22_ and T_23_ were designated as tightly bound water, weakly bound water and free water, respectively [29].

The results indicate a decrease in the peak area ratio of both the tightly and weakly bound water with increasing storage duration, whereas the peak area percentage of the free water increased, suggestive of a gradual transformation of bound water into free water. Additionally, the A_23_ values of the HBS/HBPs mixtures were observed to be lower compared to those of HBS, while the A_22_ values were higher. These findings indicate that the presence of HBPs enhanced the water retention capacity of the gels and delayed the separation of water from the gels. This may be attributed to the steric hindrance of HBPs and the binding of protein and starch chains, which limited the formation of hydrogen bonds among the starch chains and disrupted the tight association between them, thus hindering the diffusion and exudation of water from the starch paste [38]. During the retrogradation process, the formation of a more stable crystalline state through the recrystallization’s association with water molecules, along with the interaction of the double helix structure of starch with water to increase the proportion of bound water, indicates a close relationship between starch recrystallization, retrogradation and the distribution of water [39]. Compared to the gliadin and glutenin groups, the albumin and globulin groups had significantly higher A_22_ and lower A_23_ during storage, which means that there was more bound water and less free water in the system. This indicates that albumin and globulin can compete more effectively with starch molecules for water and reduce starch recrystallization, thus inhibiting starch retrogradation.

### 2.5. Gels’ Hardness

**Figure 4:**
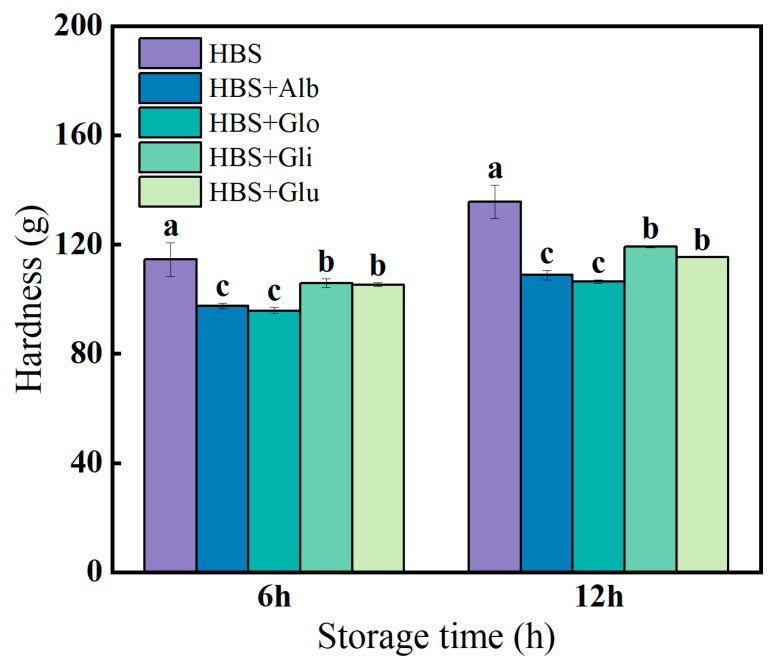
The hardness (g) of HBS and HBS/HBPs gels stored at 4 °C for 6 h and 12 h. Alb: albumin; Glo: globulin; Gli: gliadin; Glu: glutenin. The same letters represent no significant difference (*p* > 0.05), and different lowercase letters represent a significant difference (*p* < 0.05).

The most pronounced feature of retrogradation in starch-based products is the elevation of hardness, making hardness a crucial indicator for assessing the degree of starch retrogradation [23]. The hardness of the HBS and HBS/HBPs gels stored at 4 °C for 6 and 12 h is presented in Figure 4. The results indicate that the hardness increased with prolonged storage duration. In early storage, the recombination of amylose molecules through hydrogen bonds and the development of a gel network structure were the main reasons for the increase in gel hardness [39]. Compared with HBS, the addition of HBPs reduced the hardness of the gels, which was attributed to the fact that the HBPs inhibited the level of amylose rearrangement and recrystallization, resulting in too little oriented alignment of the gels to enable the formation of a strong three-dimensional network structure [40]. In addition, the lower hardness can be due to the HBPs giving the gels better water retention performance and reducing the retrogradation degree of the starch gels. The hardness of the albumin (97.48/108.84 g) and globulin (95.78/106.40 g) groups was significantly lower than that of the gliadin (105.82/119.25 g) and glutenin (105.29/115.29 g) groups at both 6 h and 12 h (*p* < 0.05). Based on the pasting properties, it could be seen that albumin and globulin inhibited pasting more significantly, and these non-swollen and unbroken starch molecules were tightly bound and restricted the movement of the starch chains, thus inhibiting the formation of gel structures [41]. In addition, albumin and globulin had a strong ability to bind water, reducing the free water volume in the gels, thereby greatly decreasing moisture loss and making the gels softer, which was consistent with the LF-NMR results [29].

### 2.6. Long-Range Ordered Structure

**Figure 5:**
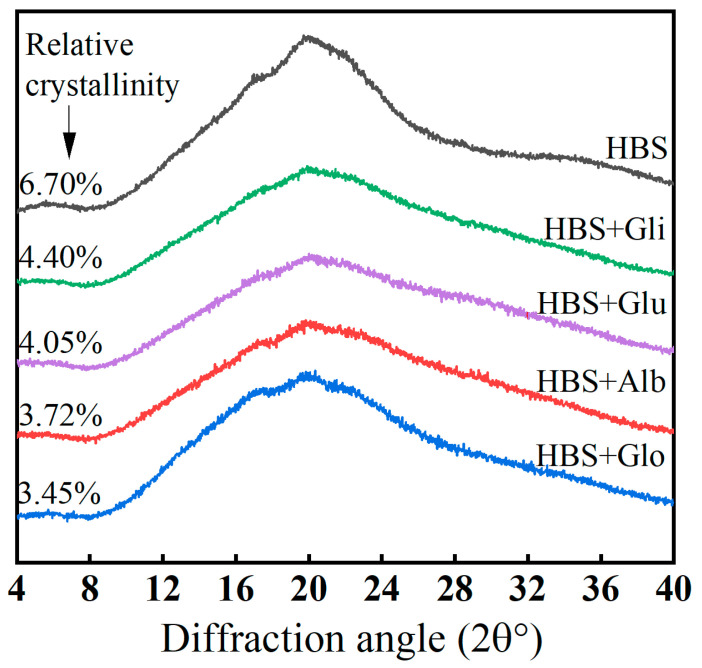
The X-ray diffraction pattern of HBS and HBS/HBPs gels stored at 4 °C for 6 h. Alb: albumin; Glo: globulin; Gli: gliadin; Glu: glutenin.

An XRD analysis can provide structural information on the microcrystalline chains of starch during retrogradation, particularly the accumulation of double helices, and the extent of starch retrogradation is commonly evaluated by measuring the relative crystallinity [42]. In Figure 5, the XRD patterns and relative crystallinity of all the samples stored at 4 °C for 6 h are presented. There was no new diffraction peak in the figure after the addition of HBPs, indicating that the HBPs did not change the crystal morphology during the storage process [22]. Two distinct diffraction peaks were observed in all the samples at 17° and 20°, representing B- and V-shaped crystal structures, respectively. Among them, V-type crystals are prominent peaks formed by the combination of lipids and amylose [39]. With the addition of HBPs, the intensity of these peaks (2θ = 20°) decreased, possibly due to the interaction of HBPs with amylose, which prevented amylose from binding to lipids and thus restraining the V-type crystals from forming [37]. The formation of B-type crystals is mainly related to the ordered structure formed after starch retrogradation, representing the degree of retrogradation [22]. During the process of retrogradation, the double helix structure, which was disrupted during gelatinization, gradually regained its order. Simultaneously, the aggregation pattern of the starch molecular chains changed, ultimately influencing the relative crystallinity of starch [43].

After storage, the addition of HBPs resulted in a decrease in the relative crystallinity of HBS, indicating that the HBPs hindered the transition of HBS from an amorphous to a polycrystalline state [22]. This might be attributed to the disruption of the original ordered gel network structure by the incorporation of HBPs, leading to a decrease in the crystalline region within the mixed systems. Consequently, HBPs can effectively delay starch retrogradation by inhibiting recrystallization. In addition, the relative crystallinity of HBS decreased by 44.48%, 48.51%, 34.33% and 39.55%, respectively, after the addition of albumin, globulin, gliadin and glutenin, which indicates that albumin and globulin could inhibit the recrystallization of HBS more effectively. This may be related to the stronger interaction between albumin and globulin and water molecules, which caused them to hinder the contact between starch molecules and free water to a greater extent, interfere with the rearrangement of starch molecules and prevent the formation of a stable double helix structure between the starch chains, thus more significantly inhibiting starch retrogradation.

### 2.7. Short-Range Ordered Structure

**Figure 6:**
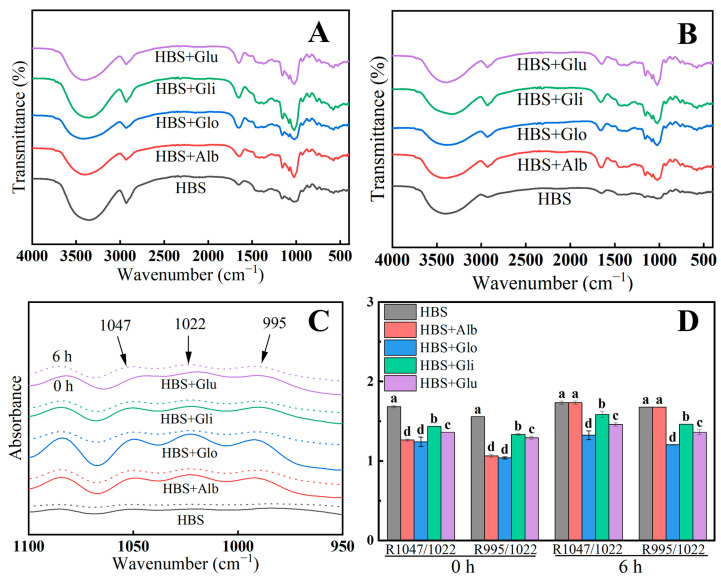
FTIR spectrum of HBS and HBS/HBPs gels stored at 4 °C for 0 h and 6 h (**A**,**B**); the deconvoluted FTIR spectra (**C**); the results of R1047/1022 and R995/1022 of HBS and HBS/HBPs gels stored at 4 °C for 0 h and 6 h (**D**). Alb: albumin; Glo: globulin; Gli: gliadin; Glu: glutenin. The same letters represent no significant difference (*p* > 0.05), and different lowercase letters represent a significant difference (*p* < 0.05).

Figure 6A,B presents the FTIR spectra of the samples that were stored at 4 °C for 0 and 6 h, respectively. It was observed that no new functional groups emerged in the spectra of the four starch/protein mixed gel systems, suggesting that the interaction between HBPs and HBS was primarily through hydrogen bonding [44]. Each sample exhibited a distinct peak in the 3000–3600 cm^−1^ spectral range, indicating the tensile vibration of O-H bonds. This peak primarily corresponds to the formation of intermolecular hydrogen bonds, and a weaker peak suggests a lower density of hydrogen bonds formed [11]. Retrogradation involves the rearrangement of starch molecules to form a three-dimensional gel network structure through hydrogen bonding, and consequently, the -OH peak can be employed as a metric to assess the degree of starch retrogradation [39]. Compared with 0 h, the absorption peaks of samples stored for 6 h were weaker, in the range of 3000–3600 cm, indicating that hydrogen bonds in the gel system were weaker after 6 h of storage [11]. Meanwhile, the samples with added HBPs had flatter bands in the 3000–3600 cm^−1^ range, especially albumin and globulin, indicating that fewer ordered crystal structures were formed during recrystallization [43]. The weak strength and limited density of the hydrogen bonds formed among the molecules were the reasons for the lower rate of retrogradation observed.

The FTIR spectra of all the samples deconvoluted, specifically in the range of 1100 to 950 cm^−1^, and are presented in Figure 6C. Previous reports suggest that the absorption peaks observed at 1047 cm^−1^, 1022 cm^−1^ and 995 cm^−1^ correspond to the ordered structure, disordered structure and double helix structure of the gel, respectively [45]. The ratio of absorbance between 1047 cm^−1^ and 1022 cm^−1^ (denoted as R1047/1022) can serve as an indicator of the degree of order in starch, while the ratio of absorbance between 995 cm^−1^ and 1022 cm^−1^ (denoted as R995/1022) reflects the presence of the double helix structure in starch [22]. As depicted in Figure 6D, a comparison of the fresh gel (0 h) with the gel stored for 6 h shows a slight increase in the values of R1047/1022 and R995/1022. This observation indicates that during storage, the starch molecules underwent rearrangement, resulting in an increase in the ordered structure. However, the R1047/1022 and R995/1022 values of the HBS/HBPs samples were lower than that of HBS, suggesting that the HBPs decreased the double helix content and suppressed the short-range ordering during starch retrogradation. Therefore, we could conclude that the addition of HBPs reduced the intermolecular hydrogen bonding and decreased the degree of ordering of the starch gel during storage, thus affecting the recrystallization process of starch and inhibiting its retrogradation.

### 2.8. SEM

**Figure 7:**
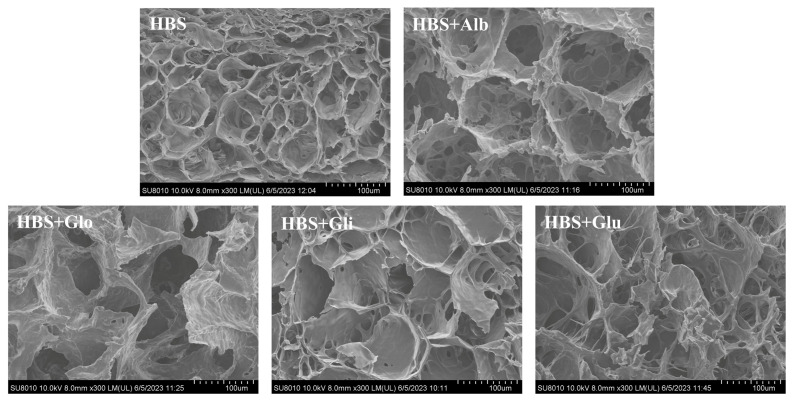
Scanning electron micrographs of HBS and HBS/HBPs gels stored at 4 °C for 6 h. Alb: albumin; Glo: globulin; Gli: gliadin; Glu: glutenin.

As shown in Figure 7, all the freeze-dried gels exhibited a similar “honeycomb” network structure, which was caused by the full development of the gel during the cooling process, and the molecules were re-aggregated to develop a tight three-dimensional net structure. In addition, the starch molecules’ rearrangement and the water distribution during refrigeration could affect the formation of gel pores [22]. Compared with the HBS gel, the HBS/HBPs gels presented a looser gel structure with larger pores. On the one hand, according to the LF-NMR results, the HBPs enhanced the percentage of bound water in the gels, suggesting that the HBPs increased the water retention of the gels. On the other hand, the HBPs could restrain the leaching of amylose and react with the leached amylose, thus restricting the cross-linking between starch molecules and impeding the amylose recrystallization, which subsequently formed a weakened gel structure. This weak gel structure could reduce the gel hardness, which is consistent with the conclusion of the hardness analysis. In addition, it could be observed that the starch gels with albumin and globulin added had larger pores and looser structures than the gels with gliadin and glutenin. Consequently, it was possible to assume that albumin and globulin have a better effect on retrogradation, which is also consistent with the results of other experiments, such as those of the pasting properties, hardness analysis and LF-NMR analysis.

## 3. Materials and Methods

### 3.1. Materials

The proteins and starches utilized in this study were sourced from HB seeds (variety Kunlun 15) cultivated in Xining, China. The total starch and amylose kits were obtained from Megazyme International Ireland Ltd., located in Wicklow, Ireland. All the reagents and chemicals utilized in the experiments were of analytical grade.

### 3.2. Extraction of Highland Barley Starch

The HB seeds were first pearled at a 20% pearling rate and then milled in a cyclone grinder to obtain highland barley flour (HBF), which was finally sieved through 100-mesh. The HBS was extracted from the abovementioned HBF by a double-enzyme method in the laboratory; the detailed method was referred to the scheme of Nie et al. [46]. The HBF (100 g) was combined with 600 mL of distilled water. Subsequently, 50 mg of cellulase and 3.08 g of xylanase were incorporated and mixed for 8 h at 50 °C using a water bath. The resulting mixture was centrifuged at 5500× *g* for 20 min, discarding all the components except for the white precipitate. The precipitate was then washed three times with deionized water and anhydrous ethanol, each time centrifuging at 5500× *g* for 20 min. After washing, the precipitate was identified as starch and dried at 25 °C for 24 h, passing through a 100-mesh sieve. Subsequently, the Megazyme kits were employed to assess the total starch content and amylose content of the extracted starch. The results indicate a total starch content of 90.59% (*w*/*w*) and an amylose content of 23.80% (*w*/*w*).

### 3.3. Extraction of Highland Barley Proteins

The four HBPs were extracted according to Tavano et al. [47]. First, defatted HBF was extracted by 20 g/L NaCl solution (1:25 *w*/*v*), stirred at 45 °C for 2 h and then centrifuged (5500× *g*, 20 min). The precipitate was used for the following extraction of gliadin and glutenin. The obtained liquid supernatant was then dialyzed in deionized water at 4 °C for 2 days. The deionized water was refreshed during the dialysis. Then, centrifugation (5500× *g*, 20 min) was performed to obtain the globulin precipitate and albumin supernatant. The supernatant was adjusted to the isoelectric point (pH 4.5) and centrifuged (5500× *g*, 20 min) to obtain the albumin precipitate.

The initial precipitate was mixed with a 75% ethanol solution, stirred at 45 °C for 2 h and centrifuged (5500× *g*, 20 min), then the supernatant was evaporated by rotation to obtain gliadin. The precipitation was mixed with a 0.1 mol/L NaOH solution, stirred at 45 °C for 2 h and centrifuged (5500× *g*, 20 min), then the supernatant precipitated glutenin at the isoelectric point (pH 4.8). Finally, the four HBPs were freeze-dried and placed at −20 °C for subsequent experiments. The method of Kjeldahl nitrogen determination was used to determine the crude protein content, and the four proteins were 80.2%, 83.2%, 86.2% and 86.1%, respectively.

### 3.4. Rapid Viscosity Analysis (RVA)

To assess the pasting properties of starch, a rapid viscosity analyzer (RVA 4500, Perten, Melbourne, Australia) was utilized. Before analysis, the four extracted HBPs were mixed with HBS in ratios of 0:100 and 10:90 (*w*/*w*). Subsequently, these mixtures (2.24 g total) were dispersed in deionized water to reach a final total weight of 28.00 g, creating a starch suspension with an 8% (*w*/*w*) consistency. The samples underwent a specific heating protocol: first, they were heated at 50 °C for 1 min, followed by a rapid increase of 12 °C per minute to reach 95 °C (held for 2.5 min), and then they were cooled to 50 °C at the same rate (held for 2 min) [48].

### 3.5. Dynamic Viscoelastic Measurements

Immediately after being prepared by the RVA, the fresh paste was transferred to a rheometer (DHR-1, TA Instruments, New Castle, DE, USA) maintained at 4 °C. The rheometer was equipped with a parallel plate system consisting of a 35 mm diameter plate with a 1 mm gap. Any excess paste around the edges was removed, and silicone oil was applied to prevent water evaporation. Before commencing the test, the samples were allowed to equilibrate at 4 °C for 10 min. Subsequently, the oscillation strain was set to 0.1%, and the oscillation frequency was adjusted to 6.28 rad/s [29]. The rheometric measurements were recorded throughout 2 h at 4 °C, capturing the evolution of the storage modulus (G′), loss modulus (G″) and loss tangent (tan δ, G″/G′) curves.

### 3.6. Differential Scanning Calorimetry (DSC)

To determine the thermal properties of the samples, a differential scanning calorimeter (PerkinElmer, Inc., Waltham, MA, USA) was utilized. Briefly, 3 mg of HBS or HBS/HBPs mixtures were placed in a crucible along with 6 μL of deionized water. The crucible was then covered and allowed to stand at 4 °C for 24 h. Subsequently, the samples were heated from 30 °C to 110 °C at a rate of 10 °C/min until complete gelatinization occurred. The gelatinization enthalpies (ΔH_g_) were noted during this process. After gelatinization, the samples were left at 4 °C for 6 h to undergo retrograde changes. They were then reheated, and the retrogradation enthalpy (ΔH_r_) was recorded [37]. The degree of retrogradation (DR) was calculated by Equation (1) [49]:

(1) $$\mathrm{DR}\;(\%)=\frac{\Delta H_{r}}{\Delta H_{g}}\;\times \;100$$

### 3.7. Low-Field Nuclear Magnetic Resonance (LF-NMR)

The migration of water during the storage of the samples was monitored by the LF-NMR analyzer (EDUMR20-015V–I, Niumag Co., Ltd., Suzhou, China) following the CPMG pulse sequence. The fresh HBS and HBS/HBPs paste (3 mL) made by RVA were piped into some 10 mL clear glass vials with screw caps and shaken to make it more homogeneous and bubble-free, and then the samples were stored at 4 °C for 0, 6 and 12 h. The spin–spin relaxation time (T_2_) and the respective peak area occupancy (A_2_) were recorded [23].

### 3.8. Hardness Analysis

The gel hardness was measured using a texture analyzer (Stable Micro Systems Ltd., Godalming, Surrey, UK) with a P/0.5 cylinder probe. The HBS and HBS/HBPs paste made by RVA were transferred to some 10 mL beakers and held at 4 °C for 6 and 12 h after cooling to room temperature. The experimental parameters were 5 g trigger force, 50% strain and the speed was 1.0 mm/s before, during and after the test [46].

### 3.9. X-ray Diffraction (XRD)

After being stored at 4 °C for 6 h, the HBS and HBS/HBPs gels were freeze-dried. An X-ray diffraction instrument (D8 Advance, Bruker, Billfeld, Germany) was then used to obtain the diffractograms of the samples [22]. The scanning range was set to 4–40° (2θ) with a scanning rate of 4°/min. MDI Jade 6 software was employed to analyze the diffraction patterns and determine the areas corresponding to the crystalline and amorphous phases. The relative crystallinity was subsequently calculated using the following formula [11]:

(2) $$\mathrm{Relative}\;\mathrm{crystallinity}\;(\%)=\frac{I_{c}}{I_{c}+I_{a}}\;\times \;100$$

where I_c_ represents the crystalline phase peak area and I_a_ represents the amorphous phase peak area.

### 3.10. Fourier-Transform Infrared Spectroscopy (FTIR)

The freeze-dried samples from Section 2.8 were mixed with KBr powder at a weight ratio of 1:100. This mixture was then thoroughly milled and pressed into flakes. Using an FTIR photometer (TENSOR 27, Billfeld, Germany), the spectra were recorded after scanning the sample 64 times with a resolution of 4 cm^−1^ over a wavelength range of 400–4000 cm^−1^. The absorbance ratios at 1047/1022 cm^−1^ and 995/1022 cm^−1^ were computed after baseline correction, smoothing and Fourier deconvolution of the 1100–950 cm^−1^ band using OMNIC 8.2 software [11].

### 3.11. Scanning Electron Microscope Analysis (SEM)

The microstructure of the samples was observed by SEM (Q45, FEI, Hillsboro, OR, USA). The gels stored at 4 °C for 6 h were freeze-dried, and their cross-sections were cut with a blade, fixed on the conductive tape of the sample table, then sputtered with gold and observed at 300× magnification at 10 kV acceleration voltage [8].

### 3.12. Statistical Analysis

All the data were presented by mean ± standard deviations from three replicates (n = 3) and analyzed by SPSS version 19.0 software. The significant differences (*p* < 0.05) were analyzed by Duncan’s test. The curve was drawn by Origin 2022 software.

## 4. Conclusions

This study shows that the water solubility of albumin and partial water solubility of globulin allowed them to compete with starch for more water, which effectively hindered the gelatinization of HBS, minimized the leaching of amylose and notably suppressed the short-term retrogradation of HBS. The formation of polymers from gliadin and glutenin upon heating adhered to the surface of starch granules, creating a physical barrier that hindered their expansion and rupture, thus preventing the rearrangement of starch chains. The pasting and thermal properties showed that HBPs enhanced the thermal stability of HBS granules, leading to incomplete starch pasting. The rise in tan δ and the drop in hardness indicate that HBS aggregation and rearrangement were restrained, implying a delay in HBS retrogradation. The XRD and FTIR findings attributed the inhibitory effect of HBPs on the relative crystallinity and double helix content of retrograded starch to the formation of hydrogen bonds between the HBPs and the hydroxyl groups of amylose, which interfered with the rearrangement of amylose. The LF-NMR analysis demonstrates that the HBPs enhanced the gels’ water-holding capacity and delayed the precipitation of water from the gels. The SEM results show that HBPS could make the gel structure looser and the void larger. In summary, HBPs can be used as a new additive to prepare degenerate-resistant HBS products, which may extend the shelf life of starchy foods and provide better texture.

## Figures and Tables

**Table 1:** Pasting parameters of HBS and HBS/HBPs samples.

Samples	PV (cP)	TV (cP)	BD (cP)	FV (cP)	SB (cP)	PT (°C)
HBS	3620 ± 86 ^a^	2273 ± 124 ^a^	1347 ± 57 ^a^	4143 ± 107 ^a^	1870 ± 23 ^a^	90.85 ± 0.57 ^b^
HBS + Alb	2670 ± 13 ^d^	1540 ± 51 ^bc^	1130 ± 64 ^c^	2708 ± 48 ^c^	1168 ± 3 ^c^	92.05 ± 1.13 ^ab^
HBS + Glo	2562 ± 33 ^e^	1521 ± 26 ^c^	1041 ± 7 ^d^	2569 ± 56 ^d^	1048 ± 30 ^d^	92.83 ± 0.04 ^a^
HBS + Gli	2806 ± 12 ^c^	1628 ± 33 ^bc^	1178 ± 45 ^b^	3058 ± 29 ^b^	1430 ± 62 ^b^	91.25 ± 0.07 ^ab^
HBS + Glu	2901 ± 10 ^b^	1657 ± 30 ^b^	1244 ± 40 ^b^	3023 ± 2 ^b^	1366 ± 32 ^b^	91.53 ± 0.46 ^ab^

Mean ± standard deviation values in the same column for each sample followed by different lowercases are significantly different (*p* < 0.05). Alb: albumin; Glo: globulin; Gli: gliadin; Glu: glutenin. PV: peak viscosity, TV: trough viscosity, FV: final viscosity, BD: breakdown, SB: setback, PT: pasting temperature.

**Table 2:** Thermal characteristic parameters and DR% of HBS and HBS/HBPs samples stored at 4 °C for 6 h.

Samples	T_O_ (°C)	T_P_ (°C)	T_C_ (°C)	ΔH_g_ (J/g)	ΔH_r_ (J/g)	DR%
HBS	61.98 ± 0.02 ^d^	65.48 ± 0.02 ^d^	68.77 ± 0.01 ^c^	8.33 ± 0.09 ^a^	0.47 ± 0.01 ^a^	5.57 ± 0.06 ^a^
HBS + Alb	62.27 ± 0.09 ^c^	65.81 ± 0.13 ^c^	68.99 ± 0.16 ^bc^	7.23 ± 0.11 ^d^	0.29 ± 0.01 ^cd^	4.03 ± 0.13 ^c^
HBS + Glo	64.83 ± 0.11 ^a^	68.49 ± 0.13 ^a^	71.81 ± 0.20 ^a^	7.67 ± 0.09 ^b^	0.27 ± 0.01 ^d^	3.41 ± 0.08 ^d^
HBS + Gli	62.23 ± 0.06 ^c^	65.72 ± 0.12 ^c^	69.53 ± 0.86 ^bc^	7.49 ± 0.02 ^c^	0.37 ± 0.01 ^b^	4.92 ± 0.16 ^b^
HBS + Glu	62.76 ± 0.06 ^b^	66.32 ± 0.06 ^b^	69.65 ± 0.11 ^b^	7.37 ± 0.14 ^cd^	0.33 ± 0.03 ^bc^	4.44 ± 0.36 ^bc^

Mean ± standard deviation values in the same column for each sample followed by different lowercases are significantly different (*p* < 0.05). Alb: albumin; Glo: globulin; Gli: gliadin; Glu: glutenin. To: the onset temperature; Tp: the peak temperature; Tc: the conclusion temperature; ΔH_g_: the gelatinized enthalpy; ΔH_r_: the retrograded enthalpy.

**Table 3:** The LF-NMR parameters of HBS and HBS/HBPs samples stored at 4 °C for 0, 6 and 12 h.

Time	Samples	T_2_ (ms)			A_2_ (%)		
		T_21_	T_22_	T_23_	A_21_	A_22_	A_23_
0 h	HBS	2.38 ± 0.41 ^a^	50.13 ± 5.22 ^bc^	541.59 ± 0.00 ^b^	0.85 ± 0.13 ^ab^	3.14 ± 0.41 ^c^	96.02 ± 0.47 ^a^
	HBS + Alb	1.77 ± 0.64 ^ab^	41.5 ± 0.00 ^c^	349.10 ± 13.83 ^d^	0.96 ± 0.03 ^a^	12.49 ± 2.03 ^a^	86.54 ± 2.05 ^b^
	HBS + Glo	0.63 ± 0.02 ^b^	52.85 ± 9.35 ^b^	357.08 ± 0.00 ^d^	0.68 ± 0.08 ^ab^	13.82 ± 2.41 ^a^	85.50 ± 2.47 ^b^
	HBS + Gli	1.59 ± 0.01 ^ab^	67.58 ± 4.69 ^a^	666.99 ± 0.00 ^a^	0.57 ± 0.04 ^b^	5.28 ± 0.29 b^c^	94.15 ± 0.33 ^a^
	HBS + Glu	1.39 ± 1.38 ^ab^	74.83 ± 2.61 ^a^	429.93 ± 17.03 ^c^	0.60 ± 0.34 ^b^	5.96 ± 0.62 ^b^	93.44 ± 0.30 ^a^
6 h	HBS	2.06 ± 0.18 ^b^	57.42 ± 2.27 ^a^	554.57 ± 22.48 ^b^	0.75 ± 0.12 ^a^	2.98 ± 0.36 ^c^	96.28 ± 0.48 ^a^
	HBS + Alb	0.73 ± 0.39 ^c^	36.21 ± 2.51 ^b^	333.13 ± 0.00 ^d^	0.78 ± 0.10 ^a^	10.95 ± 2.65 ^b^	88.26 ± 2.75 ^b^
	HBS + Glo	0.64 ± 0.00 ^c^	17.75 ± 9.76 ^c^	310.79 ± 0.00 ^e^	0.59 ± 0.07 ^ab^	13.89 ± 0.78 ^a^	85.52 ± 0.71 ^b^
	HBS + Gli	2.58 ± 0.00 ^a^	47.69 ± 0.00 ^a^	666.99 ± 0.00 ^a^	0.23 ± 0.01 ^c^	4.01 ± 1.22 ^c^	95.76 ± 1.22 ^a^
	HBS + Glu	0.92 ± 0.28 ^c^	31.15 ± 6.56 ^bc^	410.27 ± 0.00 ^c^	0.41 ± 0.34 ^bc^	5.16 ± 1.54 ^c^	94.38 ± 1.72 ^a^
12 h	HBS	2.54 ± 0.39 ^a^	61.64 ± 5.05 ^ab^	541.59 ± 0.00 ^b^	0.65 ± 0.17 ^ab^	2.35 ± 0.27 ^c^	97.34 ± 0.20 ^a^
	HBS + Alb	1.75 ± 0.61 ^abc^	32.35 ± 8.87 ^c^	333.13 ± 0.00 ^d^	0.62 ± 0.02 ^ab^	7.36 ± 1.79 ^b^	92.02 ± 1.80 ^b^
	HBS + Glo	1.05 ± 0.00 ^bc^	43.33 ± 18.78 ^bc^	318.23 ± 12.90 ^d^	0.04 ± 0.01 ^c^	11.93 ± 0.74 ^a^	88.03 ± 0.76 ^c^
	HBS + Gli	2.18 ± 1.2 ^ab^	79.51 ± 6.22 ^a^	682.98 ± 27.68 ^a^	0.92 ± 0.07 ^a^	2.56 ± 0.61 ^c^	96.52 ± 0.68 ^a^
	HBS + Glu	0.90 ± 0.25 ^c^	59.00 ± 6.83 ^b^	429.93 ± 17.03 ^c^	0.56 ± 0.37 ^b^	3.74 ± 1.83 ^c^	95.70 ± 1.81 ^a^

Mean ± standard deviation values in the same column for each sample followed by different lowercases are significantly different (*p* < 0.05). Alb: albumin; Glo: globulin; Gli: gliadin; Glu: glutenin. T_2_ and A_2_ represent the relaxation time and the ratio value of every part of the water to the total water, respectively.

## Data Availability

The data presented in this study are available in the article.

## References

[1] Obadi M., Qi Y., Xu B. (2021). Highland barley starch (Qingke): Structures, properties, modifications, and applications. Int. J. Biol. Macromol..

[2] Guo H., Lin S., Lu M., Gong J.D.B., Wang L., Zhang Q., Lin D.R., Qin W., Wu D.T. (2018). Characterization, in vitro binding properties, and inhibitory activity on pancreatic lipase of beta-glucans from different Qingke (Tibetan hulless barley) cultivars. Int. J. Biol. Macromol..

[3] Asare E.K., Jaiswal S., Maley J., Baga M., Sammynaiken R., Rossnagel B.G., Chibbar R.N. (2011). Barley grain constituents, starch composition, and structure affect starch in vitro enzymatic hydrolysis. J. Agric. Food Chem..

[4] Lian X., Kang H., Sun H., Liu L., Li L. (2015). Identification of the main retrogradation-related properties of rice starch. J. Agric. Food Chem..

[5] Lu Z.H., Donner E., Yada R.Y., Liu Q. (2016). Physicochemical properties and in vitro starch digestibility of potato starch/protein blends. Carbohydr. Polym..

[6] Anbarani N.M., Razavi S.M.A., Taghizadeh M. (2021). Impact of sage seed gum and whey protein concentrate on the functional properties and retrogradation behavior of native wheat starch gel. Food Hydrocoll..

[7] Bravo-Nunez A., Garzon R., Rosell C.M., Gomez M. (2019). Evaluation of Starch(-)Protein Interactions as A Function of pH. Foods.

[8] Zhang Y., Chen C., Chen Y., Chen Y. (2019). Effect of rice protein on the water mobility, water migration and microstructure of rice starch during retrogradation. Food Hydrocoll..

[9] Zhang B., Qiao D., Zhao S., Lin Q., Wang J., Xie F. (2021). Starch-based food matrices containing protein: Recent understanding of morphology, structure, and properties. Trends Food Sci. Technol..

[10] Guo T., Horvath C., Chen L., Chen J., Zheng B. (2020). Understanding the nutrient composition and nutritional functions of highland barley (Qingke): A review. Trends Food Sci. Technol..

[11] Kuang J., Huang J., Ma W., Min C., Pu H., Xiong Y.L. (2022). Influence of reconstituted gluten fractions on the short-term and long-term retrogradation of wheat starch. Food Hydrocoll..

[12] Goesaert H., Brijs K., Veraverbeke W.S., Courtin C.M., Gebruers K., Delcour J.A. (2005). Wheat flour constituents: How they impact bread quality, and how to impact their functionality. Trends Food Sci. Technol..

[13] Chen L., Ren F., Zhang Z., Tong Q., Rashed M.M. (2015). Effect of pullulan on the short-term and long-term retrogradation of rice starch. Carbohydr. Polym..

[14] Li J., Yuan Y., Zhang H., Zou F., Tao H., Wang N., Guo L., Cui B. (2022). Structural, physicochemical and long-term retrogradation properties of wheat starch treated using transglucosidase. Food Chem..

[15] Zeng X., Zheng B., Xiao G., Chen L. (2022). Synergistic effect of extrusion and polyphenol molecular interaction on the short/long-term retrogradation properties of chestnut starch. Carbohydr. Polym..

[16] Wang S., Li C., Copeland L., Niu Q., Wang S. (2015). Starch Retrogradation: A Comprehensive Review. Compr. Rev. Food Sci. Food Saf..

[17] Doblado-Maldonado A.F., Gomand S.V., Goderis B., Delcour J.A. (2016). The extent of maize starch crystal melting as a critical factor in the isolation of amylose via aqueous leaching. Food Hydrocoll..

[18] Ribotta P.D., Colombo A., Rosell C.M. (2012). Enzymatic modifications of pea protein and its application in protein–cassava and corn starch gels. Food Hydrocoll..

[19] Baxter G., Zhao J., Blanchard C. (2010). Albumin Significantly Affects Pasting and Textural Characteristics of Rice Flour. Cereal Chem. J..

[20] Baxter G., Blanchard C., Zhao J. (2014). Effects of glutelin and globulin on the physicochemical properties of rice starch and flour. J. Cereal Sci..

[21] Renzetti S., Arendt E.K. (2009). Effect of protease treatment on the baking quality of brown rice bread: From textural and rheological properties to biochemistry and microstructure. J. Cereal Sci..

[22] Zhou J., Jia Z., Wang M., Wang Q., Barba F.J., Wan L., Wang X., Fu Y. (2022). Effects of Laminaria japonica polysaccharides on gelatinization properties and long-term retrogradation of wheat starch. Food Hydrocoll..

[23] Zhang M., Sun C., Wang X., Wang N., Zhou Y. (2020). Effect of rice protein hydrolysates on the short-term and long-term retrogradation of wheat starch. Int. J. Biol. Macromol..

[24] Saleh M.I. (2017). Protein-starch matrix microstructure during rice flour pastes formation. J. Cereal Sci..

[25] Wan L., Wang X., Liu H., Xiao S., Ding W., Pan X., Fu Y. (2023). Retrogradation inhibition of wheat starch with wheat oligopeptides. Food Chem..

[26] Li M., Yue Q., Liu C., Zheng X., Hong J., Li L., Bian K. (2020). Effect of gliadin/glutenin ratio on pasting, thermal, and structural properties of wheat starch. J. Cereal Sci..

[27] Tarahi M., Shahidi F., Hedayati S. (2022). Physicochemical, Pasting, and Thermal Properties of Native Corn Starch–Mung Bean Protein Isolate Composites. Gels.

[28] Chen B., Zhang B., Li M.N., Xie Y., Chen H.Q. (2018). Effects of glutenin and gliadin modified by protein-glutaminase on pasting, rheological properties and microstructure of potato starch. Food Chem..

[29] Yang H., Tang M., Wu W., Ding W., Ding B., Wang X. (2021). Study on inhibition effects and mechanism of wheat starch retrogradation by polyols. Food Hydrocoll..

[30] Luo Y., Niu L., Li D., Xiao J. (2020). Synergistic effects of plant protein hydrolysates and xanthan gum on the short- and long-term retrogradation of rice starch. Int. J. Biol. Macromol..

[31] Niu L., Wu L., Xiao J. (2017). Inhibition of gelatinized rice starch retrogradation by rice bran protein hydrolysates. Carbohydr. Polym..

[32] Ito A., Hattori M., Yoshida T., Watanabe A., Sato R., Takahashi K. (2006). Regulatory Effect of Amino Acids on the Pasting Behavior of Potato Starch Is Attributable to Its Binding to the Starch Chain. J. Agric. Food Chem..

[33] Fu Z., Chen J., Luo S.-J., Liu C.-M., Liu W. (2015). Effect of food additives on starch retrogradation: A review. Starch Stärke.

[34] Liu S., Lin L., Shen M., Wang W., Xiao Y., Xie J. (2018). Effect of Mesona chinensis polysaccharide on the pasting, thermal and rheological properties of wheat starch. Int. J. Biol. Macromol..

[35] Wu C., Gong X., Zhang J., Zhang C., Qian J.Y., Zhu W. (2023). Effect of rice protein on the gelatinization and retrogradation properties of rice starch. Int. J. Biol. Macromol..

[36] Wang L., Zhang L., Wang H., Ai L., Xiong W. (2020). Insight into protein-starch ratio on the gelatinization and retrogradation characteristics of reconstituted rice flour. Int. J. Biol. Macromol..

[37] Niu H., Han Q., Cao C., Liu Q., Kong B. (2018). Short-term retrogradation behaviour of corn starch is inhibited by the addition of porcine plasma protein hydrolysates. Int. J. Biol. Macromol..

[38] Hu Y., He C., Zhang M., Zhang L., Xiong H., Zhao Q. (2020). Inhibition from whey protein hydrolysate on the retrogradation of gelatinized rice starch. Food Hydrocoll..

[39] Jia Z., Luo Y., Barba F.J., Wu Y., Ding W., Xiao S., Lyu Q., Wang X., Fu Y. (2022). Effect of beta-cyclodextrins on the physical properties and anti-staling mechanisms of corn starch gels during storage. Carbohydr. Polym..

[40] Kunyanee K., Luangsakul N. (2020). The effects of ultrasound—Assisted recrystallization followed by chilling to produce the lower glycemic index of rice with different amylose content. Food Chem..

[41] Cui M., Fang L., Zhou H., Yang H. (2014). Effects of amino acids on the physiochemical properties of potato starch. Food Chem..

[42] Matignon A., Tecante A. (2017). Starch retrogradation: From starch components to cereal products. Food Hydrocoll..

[43] Bai J., Zhang L., Jia X., Ye Q., Pei J., Song Q., Ge J., Liu X., Duan X. (2024). Multi-scale structural changes and mechanistic analysis of wheat starch gels with common proteins in short-term retrogradation at low temperature. Food Hydrocoll..

[44] Pan W., Liu W., Li J., Chen Y., Yu Q., Xie J. (2023). The role of guar gum in improving the gel and structural characteristics of germinated highland barley starch. Int. J. Biol. Macromol..

[45] Li M., Pernell C., Ferruzzi M.G. (2018). Complexation with phenolic acids affect rheological properties and digestibility of potato starch and maize amylopectin. Food Hydrocoll..

[46] Nie M., Piao C., Wang A., Xi H., Chen Z., He Y., Wang L., Liu L., Huang Y., Wang F. (2023). Physicochemical properties and in vitro digestibility of highland barley starch with different extraction methods. Carbohydr. Polym..

[47] Tavano O.L., Amista M.J.M., Del Ciello G., Rodrigues M.C.M., Bono Nishida A.M., Valadares L.A., Siqueira B.M., Gomes R., Parolini M.T., Silva Junior S.I.D. (2022). Isolation and evaluation of quinoa (Chenopodium quinoa Willd.) protein fractions. A nutritional and bio-functional approach to the globulin fraction. Curr. Res. Food Sci..

[48] Kuang J., Ma W., Pu H., Huang J., Xiong Y.L. (2021). Control of wheat starch rheological properties and gel structure through modulating granule structure change by reconstituted gluten fractions. Int. J. Biol. Macromol..

[49] Wang J., Jiang X., Guo Z., Zheng B., Zhang Y. (2021). Long-term retrogradation behavior of lotus seed starch-chlorogenic acid mixtures after microwave treatment. Food Hydrocoll..

